# Microglia Susceptibility to Free Bilirubin Is Age-Dependent

**DOI:** 10.3389/fphar.2020.01012

**Published:** 2020-07-14

**Authors:** Ana Rita Vaz, Ana Sofia Falcão, Eleonora Scarpa, Carlotta Semproni, Dora Brites

**Affiliations:** ^1^ Faculty of Pharmacy, Research Institute for Medicines (iMed.ULisboa), Universidade de Lisboa, Lisbon, Portugal; ^2^ Department of Biochemistry and Human Biology, Faculty of Pharmacy, Universidade de Lisboa, Lisbon, Portugal; ^3^ Chronic Diseases Research Centre (CEDOC), Nova Medical School, Universidade Nova de Lisboa, Lisbon, Portugal

**Keywords:** unconjugated bilirubin, young and aged cultured microglia, inflammatory-miRNAs, microglia inflammatory response, pro- and anti-inflammatory markers

## Abstract

Increased concentrations of unconjugated bilirubin (UCB), namely its free fraction (Bf), in neonatal life may cause transient or definitive injury to neurons and glial cells. We demonstrated that UCB damages neurons and glial cells by compromising oligodendrocyte maturation and myelination, and by activating astrocytes and microglia. Immature neurons and astrocytes showed to be especially vulnerable. However, whether microglia susceptibility to UCB is also age-related was never investigated. We developed a microglia culture model in which cells at 2 days *in vitro* (2DIV) revealed to behave as the neonatal microglia (amoeboid/reactive cells), in contrast with those at 16DIV microglia that performed as aged cells (irresponsive/dormant cells). Here, we aimed to unveil whether UCB-induced toxicity diverged from the young to the long-cultured microglia. Cells were isolated from the cortical brain of 1- to 2-day-old CD1 mice and incubated for 24 h with 50/100 nM Bf levels, which were associated to moderate and severe neonatal hyperbilirubinemia, respectively. These concentrations of Bf induced early apoptosis and amoeboid shape in 2DIV microglia, while caused late apoptosis in 16DIV cells, without altering their morphology. CD11b staining increased in both, but more markedly in 2DIV cells. Likewise, the gene expression of HMGB1, a well-known alarmin, as well as HMGB1 and GLT-1–positive cells, were enhanced as compared to long-maturated microglia. The CX3CR1 reduction in 2DIV microglia was opposed to the 16DIV cells and suggests a preferential Bf-induced sickness response in younger cells. In conformity, increased mitochondrial mass and NO were enhanced in 2DIV cells, but unchanged or reduced, respectively, in the 16DIV microglia. However, 100 nM Bf caused iNOS gene overexpression in 2DIV and 16DIV cells. While only arginase 1/IL-1β gene expression levels increased upon 50/100 nM Bf treatment in long-maturated microglia, MHCII/arginase 1/TNF-α/IL-1β/IL-6 (>10-fold) were upregulated in the 2DIV microglia. Remarkably, enhanced inflammatory-associated microRNAs (miR-155/miR-125b/miR-21/miR-146a) and reduced anti-inflammatory miR-124 were found in young microglia by both Bf concentrations, while remained unchanged (miR/21/miR-125b) or decreased (miR-155/miR-146a/miR-124) in aged cells. Altogether, these findings support the neurodevelopmental susceptibilities to UCB-induced neurotoxicity, the most severe disabilities in premature babies, and the involvement of immune-inflammation neonatal microglia processes in poorer outcomes.

## Introduction

Neonatal hyperbilirubinemia, or jaundice, is a common condition of the newborn, which may cause injury to neurons and glial cells, like microglia, due to increased levels of unconjugated bilirubin (UCB) (Brites, 2012). Neurological damage is produced by a small fraction of UCB that is not bound to albumin (free bilirubin, Bf) and cross the blood-brain barrier (Vodret et al., 2015). However, the molecular mechanisms of bilirubin-induced neurological dysfunction (BIND) and the determinants of cellular vulnerability and reversibility are still only partially clarified, namely those associated with microglia activation and dysfunction. The susceptibility of neonates to BIND is also far from being understood. In our previous studies we showed that immature neurons and astrocytes are more vulnerable than older ones, and that young astrocytes produce a larger amount of inflammatory cytokines (Falcão et al., 2005; Falcão et al., 2006). The effects produced are related with the nervous cell type and the intrinsic signaling pathways involved. For instance, neurons revealed to be highly vulnerable to UCB-induced cell death, while glial cells are more involved in glutamate release and neuroinflammatory-associated alterations (Brites, 2012).

Studies on microglia dysfunction and response to elevated concentrations of UCB and Bf, aside ours, are practically non-existent. We were the first to demonstrate that UCB activates and damages microglial cells (Gordo et al., 2006; Silva et al., 2010). UCB caused the switch from the ramified morphology into an amoeboid shape, first inducing phagocytosis and later on the release of inflammatory cytokines and glutamate that culminated in cell death by apoptosis and necrosis. Last evidences demonstrated that neuroinflammation is a key factor of BIND and neuro-behavioral abnormalities in a mouse model of severe neonatal hyperbilirubinemia (Vodret et al., 2018), and that exosomes from the cerebrospinal fluid of full-term newborns with acute bilirubin encephalopathy contain immune-inflammatory associated proteins (Tan et al., 2020).

Microglia are the main neuroimmune cells that provide neuroprotection by constantly surveying and sensing changes within the brain, by clearing dying neurons and pathogens, and by exerting immunoregulation. To fulfill these complex and multivariate tasks, microglia adopt distinct phenotypes (Hickman et al., 2018). Their functions are very important during the neurodevelopment period, by participating in neurogenesis and synaptic pruning programs (Cowan and Petri, 2018). It is nowadays accepted that microglia activation occurs in a continuum way and involves changes in gene expression and metabolism, cellular morphology, motility, migration, phagocytosis, proliferation, and ultimately death, as well as variations in cell-derived secretome (Prinz et al., 2019).

Recently, it was shown that the secretome from phagocytic microglia restricts neurogenesis, modulating the balance between proliferation and survival, at least in the neurogenic niche (Diaz-Aparicio et al., 2020). In addition, it was shown that the secretome from the anti-inflammatory microglia increase the survival and migration of neural stem cells, while restrains astrocyte differentiation, as compared with the pro-inflammatory phenotype (Osman et al., 2019). Upon activation, microglia release a variety of toxic and proinflammatory mediators, such as reactive oxygen and nitrogen species, cytokines, alarmins and inflammatory-associated microRNAs (inflamma-miRs) (Brites and Vaz, 2014; Sochocka et al., 2017; Guo et al., 2019; Brites, 2020). In such conditions, microglia can present multiple activation phenotypes that may co-exist due to their plasticity (Stratoulias et al., 2019). In the steady-state, the surveilling microglia express the CX3C chemokine receptor 1 (CX3CR1) for the neuronal fractalkine (CX3CL1) (Sheridan and Murphy, 2013), an axis with important role in the communication between neurons and microglia, as well as in the modulation of neuroinflammation (Corona et al., 2010; Thome et al., 2015; Bar and Barak, 2019). CX3CR1 relevance was also demonstrated in the synaptic refinement and transmission in the developing hippocampus (Paolicelli et al., 2011). Microglia activation also results from the stimulus of damaged-associated molecular pattern (DAMP) alarmins that are released from dying cells and stimulate the innate immune system (Gulke et al., 2018). One of the most important is the high mobility group box 1 (HMGB1) that, similarly to pro-inflammatory cytokines, interacts with Toll-like receptors (TLRs), among others, inducing the transcription of inflammatory mediators. HMGB1 by activating the NOD-like receptor family pyrin domain containing 3 (NLRP3) inflammasome (Chi et al., 2015) promotes the maturation and release of interleukin (IL)-1β by the activation of caspase-1 (Walsh et al., 2014).

Inflamma-miRs have a critical function in inducing microglia polarization and once in the microglia secretome have a paracrine impact on the modulation of other cell types (Alexander et al., 2015; Fernandes et al., 2018; Vaz et al., 2019; Brites, 2020). From the several inflamma-miRs, miR-155 and miR-125b usually associate with a pro-inflammatory phenotype, miR-124 with immunoregulation (Ponomarev et al., 2011; He et al., 2014), and miR-146a and miR-21 with anti-inflammatory effects and cellular senescence (Bu et al., 2017). Expression of inflamma-miRs by microglia were found to be age-dependent (Burgess et al., 2015; Huan et al., 2018). We showed that microglia isolated from the brain cortex of 2 day-old mice and kept for short- and long-term cultures, switch from an activated/reactive to senescent-like phenotypes (Caldeira et al., 2014). Increased BIND in the neonatal period may relate with a specific predisposition of the young microglia to bilirubin injury, a topic that was never investigated.

Bf neurotoxicity has been related with concentrations near or modestly above its aqueous solubility of 70 nM (Ostrow et al., 2003). Actually, Bf levels below 70 nM were shown to protect neuronal cells from oxidative damage (Barañano et al., 2002; Sedlak et al., 2009). However, we reported that concentrations of 100 and even 10 nM Bf may cause oxidative stress and injury to neocortical synaptosomes after 4 h of incubation (Brito et al., 2004). Recently, 24 h and 48 h treatment with 70 nM and 140 nM Bf compromised the cell viability of SH-SY5Y human cells and the highest concentration caused oxidative stress and DNA damage (Rawat et al., 2018). Such protective and neurotoxic distinct effects may associate to different cell types and culture models used by the researchers.

Having all the above comments in mind, in this study we evaluated how young and long-maturated mouse cortical microglia are activated/injured by Bf concentrations of 50 nM (below the limit of 70 nM) and 100 nM (slightly above the aqueous solubility), after 24 h culture. For that, we used our established primary microglial cell culture model to obtain cells with 2 and 16 days *in vitro* (DIV) (Caldeira et al., 2014; Caldeira et al., 2017). We examined microglia morphologies and markers of oxidative stress, as well as the expression of the glutamate transporter GLT-1, HMGB1, CX3CR1, and inflammatory-associated mediators, including inflamma-miRs, in each differently aged microglia treated with 50 and 100 mM Bf, to better understand the role of microglia in the increased susceptibilities of preterm and term neonates to UCB neurotoxicity.

Our data show that young (2DIV) microglia are more likely activated, with increased number of amoeboid cells and HMGB1 positive cells than long-maturated (16DIV) ones. Overexpression of GLT-1, inducible nitric oxide synthase (iNOS), inflammatory cytokines, major histocompatibility complex II (MHCII), arginase 1, and all inflamma-miRs, except miR-124, was the result of both Bf concentrations in immature cells. On the other hand, the long-maturated microglia were more irresponsive to UCB-immunostimulation. These cells just showed increased CX3CR1, depressed inflamma-miRs and upregulation of arginase 1 and IL-1β, thus revealing a restrained inflammatory phenotype upon interaction with Bf. Such understanding may be important to define therapeutic interventions to recover microglia neuroprotection in jaundiced neonates presenting an increased risk of UCB encephalopathy.

## Materials and Methods

### Animals

This study was performed in accordance with the European Community guidelines (Directives 86/609/EU and 2010/63/EU, Recommendation 2007/526/CE, European Convention for the Protection of Vertebrate Animals used for Experimental or Other Scientific Purposes ETS 123/Appendix A) and Portuguese Laws on Animal Care (Decreto-Lei 129/92, Portaria 1005/92, Portaria 466/95, Decreto-Lei 197/96, Portaria 1131/97). All the protocols used in this study were approved by the Portuguese National Authority (General Direction of Veterinary). Every effort was made to minimize the number of animals used and their suffering.

### Primary Culture of Microglia

Mixed glial cultures were prepared from 1- to 2-day-old CD1 mice as usual in our lab (Caldeira et al., 2014). Briefly, cells (4 × 10^5^ cells/cm^2^) were plated in culture medium [DMEM-Ham’s F-12 medium supplemented with 2 mM L-glutamine, 1 mM sodium pyruvate, non-essential amino acids, 10% fetal bovine serum (FBS), and 1% antibiotic-antimycotic] and maintained at 37°C in a humidified atmosphere of 5% CO_2_. After 21 days in mixed culture, microglia were isolated by mild trypsinization according to the method of Saura et al. (2003). This method resulted in the detachment of an upper layer of cells containing astrocytes, whereas microglia remained attached to the bottom of the well. The medium containing detached cells was removed and the initial mixed glial-conditioned medium was added. Culture medium was replaced every 4 days and attached microglia were maintained in culture until 2 or 16DIV, in order to have young and long-maturated cells, respectively (Caldeira et al., 2014). Purity of microglia was nearly 100% in 2DIV cells and 98% in 16DIV cells, in which astrocytes accounted for the 2% contamination when assessed by immunocytochemical staining with a primary antibody against glial fibrillary acidic protein (GFAP). Contamination by neurons was null after staining with microtubule-associated protein 2 (MAP-2). Characterization of the 2DIV and 16DIV microglia was previously described for nuclear factor kappa B (NF-κB) activation, migration ability, phagocytic capacity, together with reactive and cell senescence markers (Caldeira et al., 2014).

### Cell Treatment

Microglia with 2DIV and 16DIV were incubated during 24 h, at 37°C, in the absence (control) or in the presence of 50 and 100 nM Bf which mimic the concentrations found in moderate and severe neonatal jaundiced conditions, respectively. Such concentrations were selected considering that the aqueous saturation limit is 70 nM and that toxicity usually occurs at Bf levels near or modestly above the aqueous solubility of bilirubin (Ostrow et al., 2003). Stock Bf solutions were extemporarily prepared in 0.1 N NaOH solution, immediately before incubation and under light protection to prevent photo-degradation. Value of pH was adjusted to 7.4 by adding 0.1 N HCl. UCB was obtained from Sigma and purified as usual in our lab (Vaz et al., 2011a).

### Immunocytochemistry

We assessed microglia activation by evaluating cell morphology with Iba1 and CD11b intensity/area by immunostaining. After incubation, cells were fixed for 20 min with 4% (w/v) paraformaldehyde and incubated with primary antibodies raised against Iba1 (rabbit, 1:150, Wako) or Cd11b (rat, 1:100, Biolegend), followed by species-specific secondary antibodies. For HMGB1, we used the primary antibody against HMGB1 (mouse, 1:100, Biolegend) followed by species-specific secondary antibody. In this case, we counted the percentage of HMGB1-positive cells above a certain threshold, considering the same background and time of exposure when acquiring the image. We also evaluated glutamate transporter GLT-1 expression by immunocytochemistry. Here, we used the primary antibody raised against GLT-1 (rabbit, 1:180, Abcam) followed by species-specific secondary antibody. Data were expressed as fold change relatively to aged-matched non-treated cells (control).

In all cases, cell nuclei were stained with Hoechst 33258 dye. Fluorescence was visualized using a fluorescence microscope (model AxioScope.A1) coupled with AxioCam HR (Zeiss). Ten random fields were acquired per sample. Image analysis and processing of the fluorescent-labelled cells were done using ImageJ software, and individual microglia manually traced with this software (Cunha et al., 2016).

### Quantification of Nitrite Levels

Levels of nitric oxide (NO) were estimated by measuring the concentration of nitrites (NO_2-_), a product of NO metabolism, in the supernatants of microglial cells (Vaz et al., 2011a). Extracellular media, free from cellular debris, was mixed with Griess reagent [1% (w/v) sulfanilamide in 5% H_3_PO_4_ and 0.1% (w/v) N-1 naphtylethylenediamine, from Sigma-Aldrich, in a proportion of 1:1 (v/v)] in 96-well tissue culture plates for 10 min in the dark, at room temperature. The absorbance was red at 540 nm using a microplate reader (Bio-Rad Laboratories, Hercules, CA, USA) and a calibration curve used in each assay. Results were expressed as fold change relatively to aged-matched non-treated cells (control).

### Mitochondria Fluorescence Intensity

The mitochondrial-related oxidative activity was detected by Mitotracker^®^ Red CMXRo (Invitrogen), a soluble dye that permeates live cells in the not-fluorescent form and, once inside the cells, is oxidized and converted into the fluorescent form, thus staining active mitochondria (Barateiro et al., 2012). After incubation, cells were incubated with 100 nM MitoTracker dye, under light protection, for 30 min at room temperature. Cells were then fixed for 20 min with paraformaldehyde 4% in PBS. Cell nuclei were stained with Hoechst 33258 dye (1:1000) and the relative fluorescence was visualized by fluorescence microscope. Ten fields were captured randomly and analysed by using the ImageJ software. The number of nuclei was counted and the average MitoTracker intensity per cell calculated. Results were then expressed as fold change relatively to aged-matched non-treated cells (control).

### Quantitative Real-Time PCR

Quantitative Real-Time (qRT)-PCR was performed as usual in our lab (Vaz et al., 2019). Briefly, total RNA was extracted from microglia using TRIzol^®^ (LifeTechnologies), according to manufacturer’s instructions. Total RNA was quantified using Nanodrop ND-100 Spectrophotometer (NanoDrop Technologies) and conversion to cDNA was performed with GRS cDNA Synthesis Master Mix (GRiSP, Porto, Portugal). qRT-PCR was performed on a QuantStudio 7 Flex Real-Time PCR System (Applied Biosystems) using an Xpert Fast Sybr Blue (GRiSP). qRT-PCR was accomplished under optimized conditions: 50°C for 2 min followed by 95°C for 2 min and finally 50 cycles at 95°C for 5 s and 62°C for 30 s. In order to verify the specificity of the amplification, a melt-curve analysis was performed, immediately after the amplification protocol. Non-specific products of PCR were not found in any case. Results were normalized to β-actin and expressed as fold change. The sequences used for primers are indicated in the **Supplementary Table 1**. For miRNA analysis, conversion of cDNA was achieved with the universal cDNA Synthesis Kit (#203301, Exiqon), following manufacturer’s recommendations and as usual in our lab (Cunha et al., 2016). The Power SYBR^®^ Green PCR Master Mix (Applied Biosystems) was used in combination with predesigned primers (Exiqon), indicated in the **Supplementary Table 1**, using SNORD110 as the reference gene. The reaction conditions consisted of polymerase activation/denaturation and well-factor determination at 95°C for 10 min, followed by 50 amplification cycles at 95°C for 10 s and 60°C for 1 min (ramp-rate 1.6°/s). Relative mRNA and miRNA concentrations were calculated using the ΔΔCT equation. All samples were measured in duplicate. Results were expressed as fold change relatively to aged-matched non-treated cells (control).

### Determination of Cell Death

The rates of cell viability were obtained by Guava Nexin flow cytometry (Guava easyCyte 5HT flow cytometer) that differentiated three cell populations: viable cells (annexin V-PE and 7-AAD negative), early-apoptotic cells (annexin V-PE positive and 7-AAD negative) and late-apoptotic/necrotic cells (annexin V-PE and 7-AAD positive). In brief, after incubation with trypsin for 5 min at 37°C, the cells were collected and joined to the incubation media. Then the samples were precipitated and re-suspended in PBS (with BSA 1%). Guava Nexin^®^ Reagent was added to the samples during 20 min in darkness, following manufacturer’s instructions and as usual in our lab (Gomes et al., 2018).

### Statistical Analysis

Results from at least three different independent experiments, performed in duplicate, were expressed as mean ± SEM. For each time in culture (2DIV or 16DIV), comparisons between non-treated microglia (control) and Bf-treated (50 nM and 100 nM) were performed by one-way ANOVA followed by multiple comparisons Bonferroni post-hoc correction using GraphPad Prism 7 (GraphPad Software, San Diego, CA, USA). P-values of 0.05 were considered statistically significant.

## Results

### Young Microglia Are Particularly Susceptible to Bf-Induced Activation and Sickness Response

We have previously demonstrated that cortical microglia isolated from 1- to 2-day-old mice pups, maintained 21 days in mixed culture with astrocytes, separated by mild trypsinization and cultured for only 2DIV show a reactive phenotype (Caldeira et al., 2014). However, such profile changed to a mature phenotype at 10DIV and to a less responsive and senescent-like microglia at 16DIV (e.g. reduced phagocytic and migration abilities, together with increased senescent-associated β-galactosidase activity and miR-146a levels). Here, we evaluated how such age-differentiated 2DIV and 16DIV microglia react to 50 nM and 100 nM Bf, considering that the first is below its aqueous saturation limit of 70 nM and the later above, with propensity to induce BIND (Ostrow et al., 2003). These concentrations allowed us to use Bf without the need of having albumin in the milieu, a condition that better mimics the *in vivo* conditions once albumin is not present in the brain, unless there is compromise of the blood-brain barrier. Moreover, though previously indicated to prevent UCB-induced toxicity towards neurons and astrocytes in mature and immature rat hippocampal slices (Dani et al., 2019), albumin was also reported to activate microglia and cause neurotoxicity (Hooper et al., 2009), what could compromise the effects of Bf by itself.

We first assessed whether Bf was able to change microglia viability. As indicated in **Table 1**, though no significant alterations were produced in the number of viable cells, both Bf concentrations increased the percentage of 2DIV cells suffering early apoptosis (6 and 7% *vs.* 3% in controls) and that of 16DIV presenting late apoptosis (between 8 and 9% *vs.* 3% in controls), indicating different cell integrity compromise by Bf depending from the time in culture. It should be noted, however, that in any circumstance cell death relatively to controls surpassed the value of 10%, attesting the validity of the model to assess microglia functional differences upon Bf treatment.

**Table 1:** Percentage of viable, early apoptotic, and late apoptotic/necrotic microglia populations before and after treatment with free bilirubin concentrations.

	2 DIV			16 DIV		
	Viable	Early apoptosis	Late apoptosis and necrosis	Viable	Early apoptosis	Late apoptosis and necrosis
**Control**	91.3 ± 1.5	3.2 ± 0.4	3.8 ± 0.5	90.4 ± 1.6	3.8 ± 0.4	3.2 ± 0.2
**Bf 50 nM**	87.6 ± 1.0	6.6 ± 1.2*	6.5 ± 1.0	87.4 ± 0.7	3.9 ± 1.0	8.3 ± 1.3*
**Bf 100 nM**	88.6 ± 1.2	5.6 ± 0.7*	7.0 ± 1.2	86.5 ± 0.8	3.9 ± 0.6	8.8 ± 0.7*

Microglial cells were treated at 2 and 16 days-in-vitro (DIV) with free bilirubin (Bf) at 50 and 100 nM, during 24 h. After incubation, the percentage of viable, early apoptotic, and late-apoptotic/necrotic cells were determined by flow cytometry with phycoerythrin-conjugated annexin V (annexinV-PE) and 7-amino-actinomycin D (7-AAD). The three populations were distinguished as follows: viable cells (annexin V-PE and 7-AAD negative), early apoptotic cells (annexinV-PE positive and 7-AAD negative), and cells in late stages of apoptosis or necrosis (annexinV-PE and 7-AAD positive). Results are mean± SEM from at least 3 independent experiments performed in duplicate. *p<0.05 vs. respective control.

**Figure 1:**
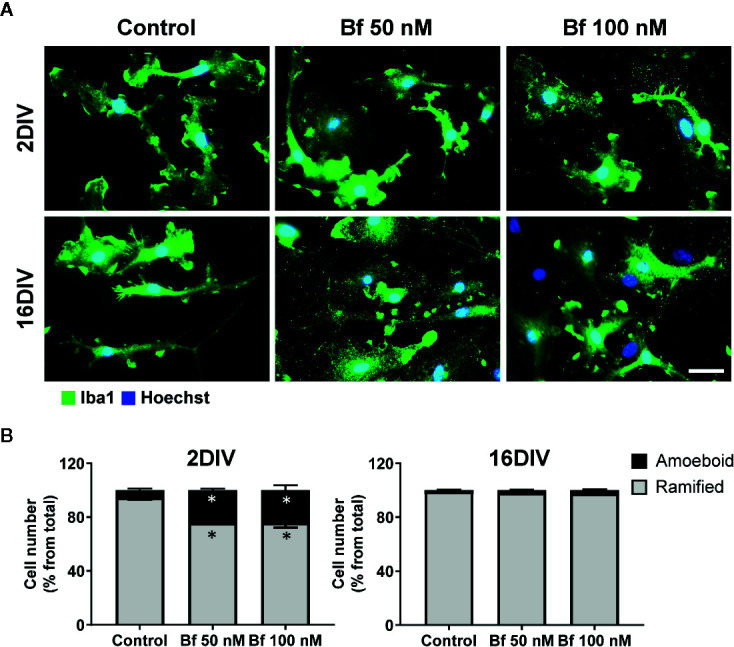
2 DIV microglia develop an amoeboid morphology upon incubation with free bilirubin (Bf), an effect not produced in the 16 DIV aged cells. Microglial cells were treated at 2 and 16 days-in-vitro (DIV) with 50 nM and 100 nM Bf for 24 h. After incubation, morphological analysis was performed by immunocytochemistry using anti-Iba1, as indicated in methods. Representative results of one experiment are shown **(A)**. Percentage of cells with ramified and amoeboid morphologies were quantified **(B)**. Scale bar represents 40 μm. Results are mean ± SEM from at least 3 independent experiments performed in duplicate. *p < 0.05 *vs.* respective controls (untreated cells).

**Figure 2:**
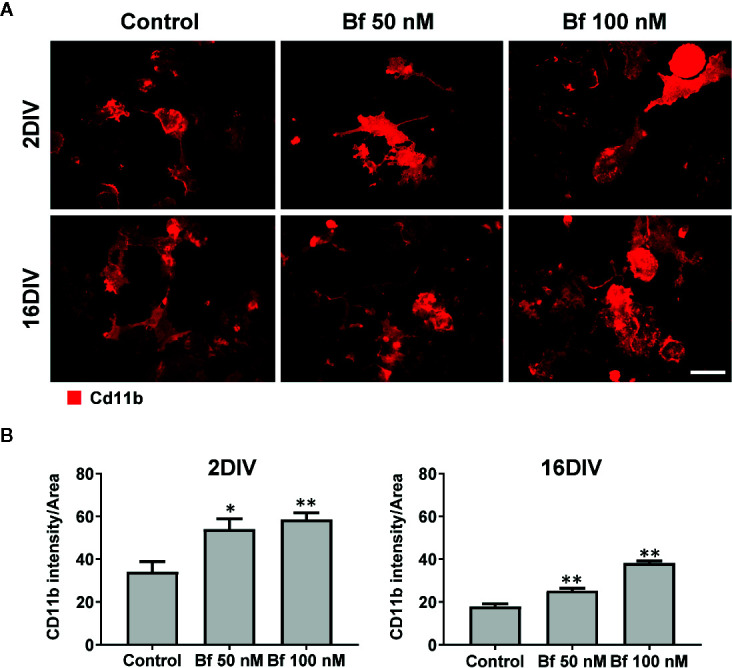
Expression of Cd11b increases in microglia after incubation with free bilirubin (Bf), though more notoriously in young 2DIV cells. Microglial cells were treated at 2 and 16 days-in-vitro (DIV) with 50 nM and 100 nM Bf for 24 h. After incubation, analysis of Cd11b intensity was performed by immunocytochemistry using anti-CD11b, as indicated in methods. Representative results of one experiment are shown **(A)**. Quantification of the fluorescence was performed by determining the mean fluorescence intensity of each cell and normalized to their total area **(B)**. For that, individual cells were manually traced using ImageJ software. Scale bar represents 40 μm. Results are mean ± SEM from at least 3 independent experiments performed in duplicate. *p < 0.05 and **p < 0.01 *vs.* respective controls (untreated cells).

**Figure 3:**
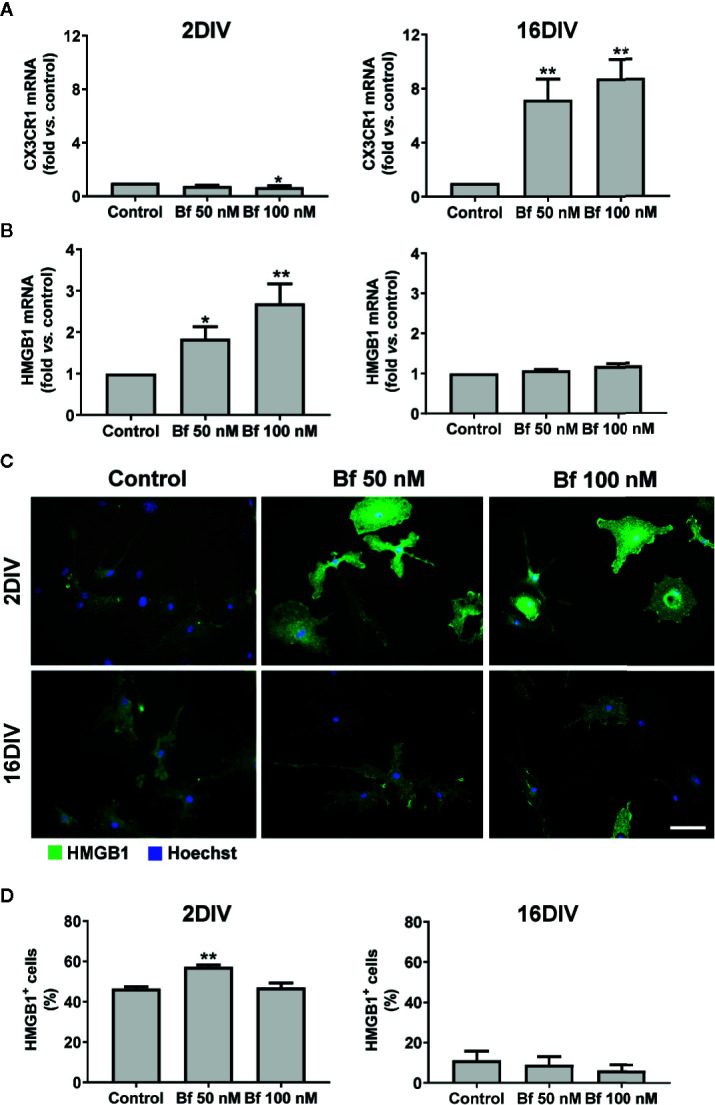
Activation of 2DIV microglia with free bilirubin (Bf) is associated with decreased CX3CR1 and increased HMGB1 levels, which are not observed in 16DIV cells. Microglial cells were treated at 2 and 16 days-in-vitro (DIV) with 50 nM and 100 nM Bf for 24 h. After incubation, CX3CR1 **(A)** and HMGB1 **(B)** gene expression was evaluated by Real Time-PCR, as specified in methods. HMGB1 protein detection was performed by immunocytochemistry using anti-HMGB1, as indicated in methods. Representative results of one experiment are shown **(C)**. Percentage of HMGB1 positive cells was performed using ImageJ software and applying a threshold **(D)**. Results are fold change **(A, B)** and mean percentage ± SEM (D) *vs.* respective control (untreated cells) from at least 4 independent experiments performed in duplicate. *p < 0.05 and **p < 0.01 *vs.* respective controls.

Microglia activation is usually associated with rounded/amoeboid shapes, while the ramified morphology relates with the surveillant state (Graeber, 2010). Bf-stimulation of microglia morphological alterations after 24 h incubation was assessed by immunofluorescent labelling against Iba1. While the Bf-treated 16DIV cells remained ramified (almost 100%), an increased number of amoeboid cells was observed in the 2DIV microglia (**Figure 1**), supporting the acquisition of a more activated/reactive stage (Domercq et al., 2013). To note that the effects were similar with either 50 or 100 nM of Bf (p<0.05). We hypothesize that such result derives from the soluble fraction interacting with the microglia plasmatic membrane, i.e., the Bf soluble species. Evaluation of CD11b immunostaining, a β-integrin marker associated with microglial activation during neurodegenerative inflammation (Roy et al., 2006), further validated the preferential activation of 2DIV cells relatively to 16DIV microglia (**Figure 2**). As a proof of concept for the increased reactivity in younger microglia, we evaluated the constitutive “calming” microglial signaling marker CX3CR1. Indeed, CX3CR1 expression has been associated with a steady state/surveilling microglia and linked to the neuronal CX3CL1 signaling in the healthy brain (Eyo and Wu, 2013). In **Figure 3A**, it is evident that 100 nM Bf reduces the expression of CX3CR1 in 2DIV cells (p<0.05), which is consistent with the activated profile previously observed in the young microglia. In contrast, CX3CR1 gene expression was induced by both Bf concentrations in 16DIV cells, thus accounting for their supportive behavior when in the presence of Bf.

The alarmin HMGB1 can be released under pathological conditions and initiate inflammatory cascades. In our model, we observed a marked elevation of HMGB1 gene expression in the young cells exposed to Bf 50 nM and to 100 nM, while no differences were found in the long-maturated cells (**Figure 3B**). Since the effects of HMGB1 are dependent on complex formation with different ligands that usually activate the innate immune system and promote inflammation (Sha et al., 2008; Youn et al., 2008), we additionally evaluated if the localization of the HMGB1 protein was cytoplasmic or nuclear, and quantified the number of HMGB1-positive cells by immunofluorescence (above a common threshold). As depicted in **Figures 3C, D**, HMGB1 was found present in both nucleus and cytoplasm, with an increased number of 2DIV positive microglia upon interaction with 50 nM Bf (p<0.05), relatively to the non-treated cells (control). In contrast, 16DIV microglia behaved as non-responders to the Bf stimulus.

**Figure 4:**
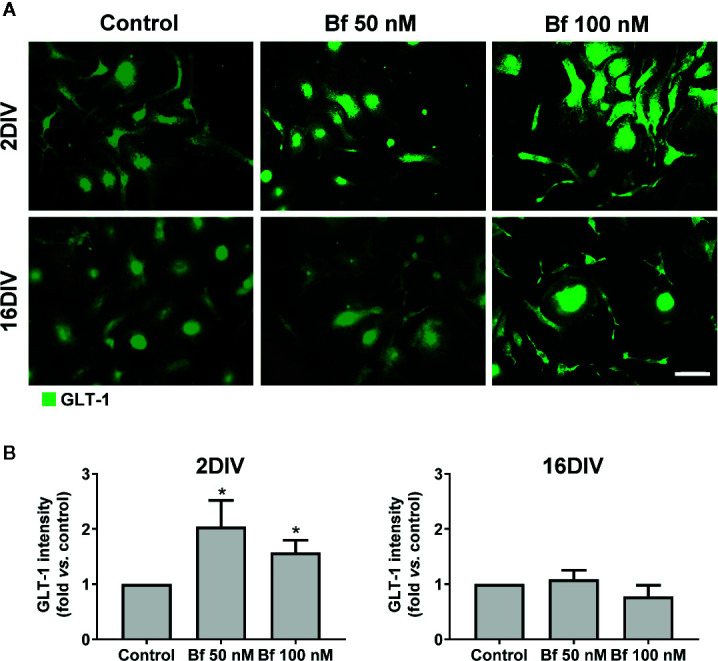
Free bilirubin (Bf) upregulates GLT-1 only in young microglia. Microglial cells were treated at 2 and 16 days-in-vitro (DIV) with 50 nM and 100 nM Bf for 24 h. After incubation, detection of GLT-1 levels was performed by immunocytochemistry using anti-GLT-1, as indicated in methods. Representative results from one experiment are shown **(A)**. Scale bar represents 40 μm. Quantification of the mean fluorescence intensity of GLT-1 in each cell was normalized to their total area **(B)**. For that, individual cells were manually traced using ImageJ software. Results are mean ± SEM fold change *vs*. respective controls (untreated cells) from at least 5 independent experiments performed in duplicate. *p < 0.05 *vs.* respective control.

To further explore the activation of 2DIV microglia by Bf, we next explored the expression of GLT-1, since it was shown to be highly expressed by activated microglia after facial nerve axotomy (Lopez-Redondo et al., 2000), or subsequently to LPS stimulation and tumor necrosis factor-alpha (TNF-α) increase (Persson et al., 2005). Such upregulation of GLT-1 was suggested to be a mechanism to prevent glutamate excitotoxicity (Lai et al., 2013), which contributes to neuronal damage during neuroinflammation (Barger et al., 2007). As expected, GLT-1 expression by immunocytochemistry was upregulated by both concentrations of Bf in the 2DIV microglia (p<0.05), contrasting with no changes in the long-maturated cells (**Figure 4**). These results reinforce the ability of Bf to activate the young microglia and indicate that the long-matured cells acquire resistance to the immunostimulant effects of Bf.

### Elevated Levels of Bf Only Accelerate Mitochondrial-associated Nitric Oxide Stress in 2DIV Cells, Though Upregulate iNOS Gene Expression in Both Young and Aged Microglia

**Figure 5:**
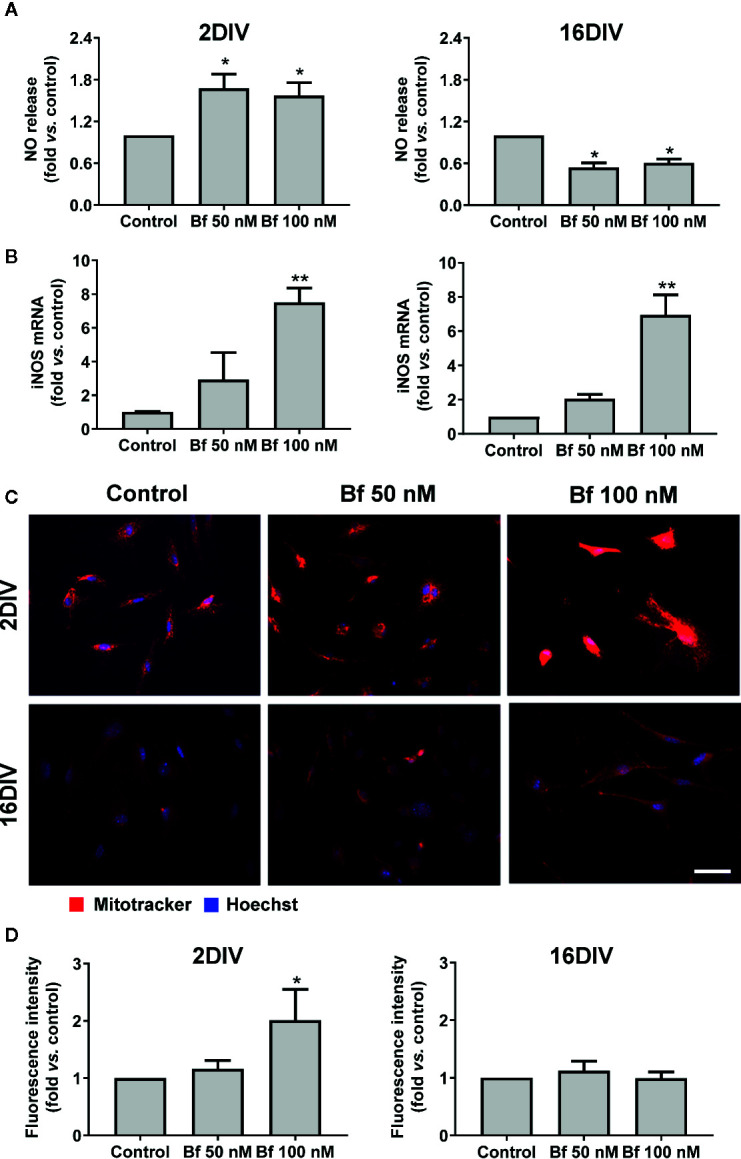
High levels of free bilirubin (Bf) upregulate iNOS gene expression in the differently aged microglia, though mitochondrial-associated NO stress only occurs in 2DIV cells. Microglial cells were treated at 2 and 16 days-in-vitro (DIV) with 50 nM and 100 nM Bf for 24 h. After incubation, NO levels **(A)** were assessed by Griess reagent and iNOS mRNA **(B)** by Real Time-PCR. Mitochondria was stained with mitotracker red and representative results from one experiment are shown **(C)**. Fluorescence intensity values were obtained by using ImageJ software to define the integrated density **(D)**. Results are mean± SEM fold change *vs*. respective controls (untreated cells) from at least 4 independent experiments performed in duplicate *p < 0.05 and **p < 0.01 *vs.* respective controls.

Taking into account the activation profile induced by Bf and because oxidative stress is a central regulator of HMGB1’s translocation, release, and activity in inflammation and cell death (Yu et al., 2015), we further assessed nitrosative stress-related mediators in our model. As depicted in **Figure 5A**, a clear elevation of NO production was observed in 2DIV Bf-treated microglia (p<0.05) with concentration-independent levels. In contrast, the 16DIV cells showed a reduction (p<0.05), which was again similar for 50 nM and 100 nM Bf. Data reinforce the age-dependent response of microglia to these concentrations of Bf in terms of NO production. However, when we evaluated the mRNA gene expression of inducible nitric oxide synthase (iNOS), we verified that both 2DIV and 16DIV exhibited elevated levels when treated with Bf 100 nM (p<0.01, **Figure 5B**). Equally, increased gene expression of iNOS in the same model and independently of the microglia age in culture was obtained upon amyloid-β exposure (Aβ) (Caldeira et al., 2017).

To assess whether the NO release resulted from an increase in microglial mitochondrial activity, we have used MitoTracker^®^ Red, a cell-permeant probe that enters in actively respiring cells, where it is oxidized and sequestered in the mitochondria, reacting with thiols of proteins/peptides and forming an aldehyde-fixable conjugate. Higher staining densities of this probe was present in 2DIV cells treated with Bf 100 nM (p<0.05, **Figures 5C, D**), while changes were absent in 16DIV cells, suggesting that only the younger cells increase mitochondrial-related NO stress upon Bf stimulation. Therefore, we may hypothesize that the elevation of iNOS by an age-independent Bf stimulation can result from other simultaneous intracellular activated signaling pathways not involving mitochondria (Aktan, 2004).

### Upregulation of Transcriptional Inflammatory Pathological Profiles Is a Specific Feature of Bf-Treated Young Microglia

Having observed that 2DIV microglia show a higher susceptibility to activation by Bf than the long-maturated cells, we next determined the differential expression of polarized-specific genes in Bf-treated cells. In our previous studies the use of genes associated with anti-inflammatory and pro-inflammatory microglia allowed the categorization of microglia polarized state subpopulations (Silva et al., 2010; Silva et al., 2011), including in the 2DIV/16DIV model here used (Caldeira et al., 2014; Caldeira et al., 2017).

**Figure 6:**
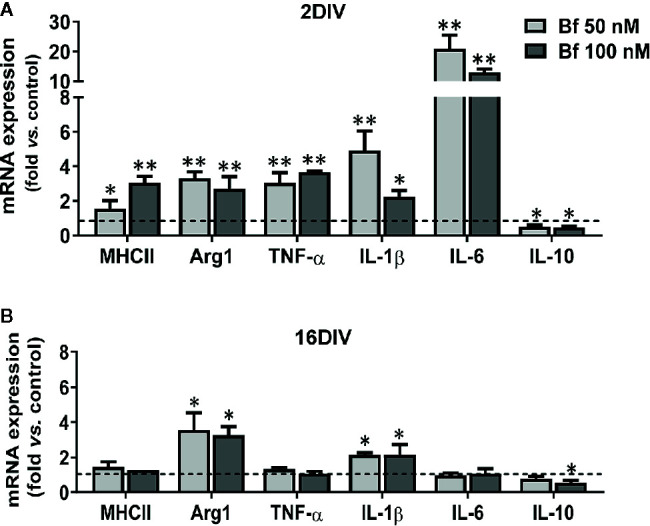
Young microglia treated with high levels of free bilirubin (Bf) show a prevalent upregulation of transcriptional inflammatory mediators, which are marginally exhibited by 16DIV cells. Microglial cells were treated at 2 and 16 days-in-vitro (DIV) with 50 nM and 100 nM Bf for 24 h. After incubation, mRNA expression of MHCII, Arginase 1 (Arg1), TNF-α, IL-1β, IL-6, and IL-10 was determined by Real Time-PCR in 2 DIV **(A)** and 16 DIV **(B)** cells. Results are mean± SEM fold change *vs*. respective controls (untreated cells) from at least 5 independent experiments performed in duplicate. *p < 0.05 and **p < 0.01 *vs.* respective controls.

As shown in **Figure 6**, arginase 1 and IL-1β were equally elevated in 2DIV and 16DIV microglia after treatment with Bf (p<0.01 and p<0.05 in the first and p<0.01 in 2DIV cells, p<0.05 in 16DIV ones for the later, for both Bf concentrations). Also included in the similar transcriptional profile induced by Bf-stimulation in young and aged microglia is the decrease of the anti-inflammatory IL-10 (p<0.05 for both Bf concentrations in the former and only for Bf 100 nM in the later). However, the young activated microglia were unique in additionally displaying upregulated transcriptional profiles of MHCII, TNF-α and IL-6 (more than 10-fold), supporting the increased proinflammatory population subtypes generated by Bf stimulation in young cells relatively to the older ones that revealed to be poor responders.

**Figure 7:**
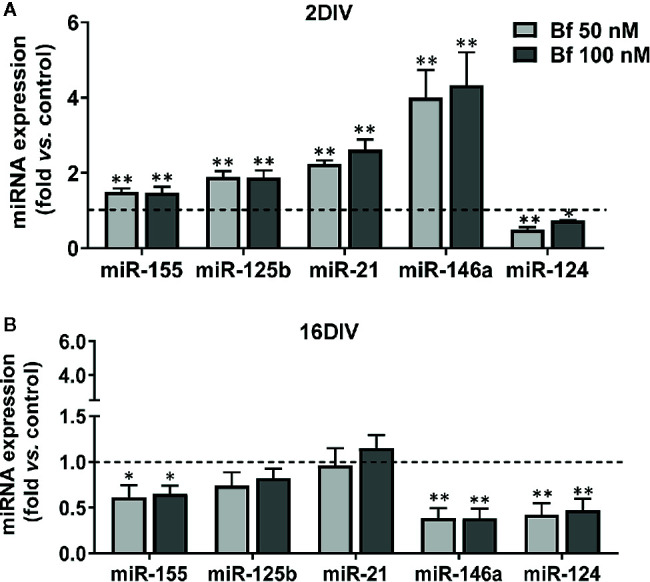
Young microglia treated with high levels of free bilirubin (Bf) show upregulation of miRNAs that mediate neuroinflammation, an effect inversely sensed by the long-maturated cells. Microglial cells were treated at 2 and 16 days-in-vitro (DIV) with 50 nM and 100 nM Bf for 24 h. After incubation, expression of miR-155/-125b/-21/-146a/124 was determined by Real Time-PCR, either in 2 DIV **(A)** or 16 DIV **(B)** cells. Results are mean± SEM fold change *vs*. respective controls (untreated cells) from at least 5 independent experiments performed in duplicate. *p < 0.05 and **p < 0.01 and *vs.* respective controls.

Though there is still an incomplete clarification of the role of miRNAs in microglia, either in health or in neuropathological conditions, the dysregulation of certain miRNAs, such as inflamma-miRs, are known to contribute to abnormal cell polarization, hyper-activation and persistent neuroinflammation (Fernandes et al., 2018; Guo et al., 2019). Validating the Bf-induced signature of the common inflammatory mediators previously found in 2DIV microglia, almost all the assessed inflamma-miRs were overexpressed in young cells, but downregulated in the long-cultured microglia, independently of the concentrations (**Figure 7**). Taking into account the 3 miRNAs more involved in microglia inflammatory response, miR-155, miR-146a, and miR-124 (Su et al., 2016), the first two were upregulated in 2DIV microglia by Bf, while were depressed in the 16DIV microglia, thus reinforcing the irresponsive character of the aged microglia towards Bf. Relatively to the anti-inflammatory miR-124, it was decreased in both, this time reflecting a common deficient microglia reparative action upon Bf, independently of the age in culture (**Figure 7**). Now, if we consider the additional increase of miR-125b and miR-21 in the 2DIV Bf-treated microglia, these results additionally suggest that both anti-inflammatory and pro-inflammatory subtypes are produced by Bf originating mixed activated populations.

Overall, our results point that the susceptibility of young/activated microglia to Bf-induced inflammatory response may associate to the damaging effects of hyperbilirubinemia during the neurodevelopment period and may be implicated in poorer outcomes of the prematurely born neonates, highlighting the need of immunoregulatory strategies against BIND in the sickest infants.

## Discussion

In a previous work, we developed an experimental method to naturally age microglia after isolation from the cortex of neonatal mice brains (Caldeira et al., 2014). We showed that these cells presented markers of a reactive phenotype early in culture (2DIV) and that such profile changed along time (until 16DIV) to a less responsive microglia showing senescence biomarkers and miRNAs of a deactivated/irresponsive subtype. More recently, we demonstrated that 16DIV microglia are poorer responders to amyloid-beta (Aβ) peptide stimulation, when compared to 2DIV cells, showing reduced phagocytosis, migration ability and inflamma-miR expression (Caldeira et al., 2017). UCB/Bf have been associated to either anti-oxidant or neurotoxic properties in nerve cells depending on their concentrations (Doré and Snyder, 1999; Doré et al., 1999). Moreover, a wide spectrum of neuropathological findings by Bf in neonates and in experimental models have been documented (Wennberg et al., 1979; Cashore, 1990; Ostrow et al., 2004; Brites, 2012; Cayabyab and Ramanathan, 2019). Age-dependent pattern of susceptibilities to BIND have been successively also demonstrated in neural cells and mice models (Falcão et al., 2005; Falcão et al., 2006; Bortolussi et al., 2014). However, only few studies addressed the harmful effects of UCB/Bf in microglia function (Gordo et al., 2006; Silva et al., 2010), and none investigated whether differences in young and adult/aged microglia to Bf insults may account to the increased vulnerabilities of neonates to hyperbilirubinemia. Thus, the present work was designed to evaluate the response of young *vs.* aged microglia to Bf stimulus at concentrations mimicking moderate to high increased associated risks. The data presented here support the concept that Bf induces differential transcriptional profiles in short- and long-cultured microglia (young/aged cells) with a predominant activation and inflammatory profile manifested by the young microglia.

Modification of microglial morphology is one of the hallmarks of cell activation and polarization (Chew et al., 2006; Lynch, 2009). For that reason, we started by assessing the morphological features of microglial cells upon Bf incubation. Our first results based on Iba1 staining indicate that microglia are preferentially activated by Bf at 2DIV, as compared with 16DIV cultures, with an increased number of cells with an amoeboid shape, as well as increased CD11b stainning. Similar profiles were obtained in microglia isolated from Wistar rats at 1- to 2-day-old and treated with elevated concentrations of UCB (Silva et al., 2010) and in the Gunn rat, a model of bilirubin encephalopathy (Liaury et al., 2012). Rather than the originally-perceived all or-none response, it is now accepted that microglia are plastic cells adopting diverse phenotypes when activated (Luo and Chen, 2012). Considering that the ramified morphology is characteristic of surveillant microglia, while the amoeboid shape predominates in the activated-state (Graeber, 2010), our results show an increased number of activated cells by Bf among the young cultured microglia population. Increased susceptibility of young/immature astrocytes and neuronal cells to UCB, as compared to maturated cells, was previously evidenced (Falcão et al., 2005; Falcão et al., 2006). Early apoptosis was mainly produced in young cells while late apoptosis/necrosis was observed in the aged cells, suggesting a different balance of negative and positive factors in age-associated cell dysfunction (Tower, 2015). Such findings strongly suggest that Bf-induced toxicity develops near (50 nM) or slight above (100 nM) the aqueous saturation limit of 70 nM, a range in which UCB monomers, oligomers, and metastable small colloids are likely present (Ostrow et al., 2003). When we further characterized the expression of cell markers associated to microglia differential polarized states, we observed a decrease of the cell “calming” signal CX3CR1 in the 2DIV microglia, probably accounting for their preferential activated profile upon Bf interaction. CX3CR1-deficient activated microglia were previously associated to increased phagocytosis (Lee et al., 2010), deficient synaptic integration and emotional behavioral alterations in mice (Bolos et al., 2018), as well as to impaired cognitive function *via* increased IL-1β (Rogers et al., 2011). Excessive microglia phagocytosis of synapses and neurons is implicated in diverse pathological processes (Vilalta and Brown, 2018). Once CX3CL1-CX3CR1 axis is an important pathway to promote synaptic plasticity processes (Sheridan and Murphy, 2013), the increased levels of microglia CX3CR1 only in 16DIV after Bf exposure may reflect an adaptive cell response to promote neuronal-microglial communication. Nevertheless, elevated levels of CX3CR1 and BDNF in microglia were also associated with autism spectrum disorders (Edmonson et al., 2014). In fact, both protective and detrimental effects have been reported for CX3CR1 deletion (Gyoneva et al., 2019) and the consequences of low and high CX3CR1 expression in 2DIV and 16DIV Bf-treated microglia should be later explored in co-cultures of neurons and microglia.

The microglial “alarming” signal HMGB1 is known to function as a proinflammatory cytokine and to cause neurodegeneration, being suggested as a therapeutic target (Paudel et al., 2018). Actually, HMGB1 released from inflamed microglia was shown to bind to Mac1 (macrophage antigen complex 1) and activate the NF-κB pathway, as well as the NADPH oxidase, thus stimulating the production of multiple inflammatory and neurotoxic factors (Gao et al., 2011). Considering the association of HMGB1 with such neuroinflammatory events we anticipated its upregulation mainly in the young Bf-treated microglia. Indeed, we observed increased transcriptional profile, higher levels of cytoplasmatic HMGB1 and enhanced HMGB1-positive cells in 2DIV Bf-treated microglia, but no changes in aged ones. Such profile agrees with previous studies showing that HMGB1 and the downstream cellular inflammatory response are enhanced in young, relatively to adult or old mice (Prasad and Thakur, 1990; Webster et al., 2019). Due to its role in sustaining inflammation and causing chronic neurodegeneration (Mittal et al., 2014), as well as in mediating the process of fetal and neonatal tissue injury (Buhimschi et al., 2009), HMGB1 targeting may reveal a promising therapeutic strategy to prevent BIND and outcomes beyond the inflammatory response in neonates.

During the neuroinflammatory process, microglia release glutamate that causes excitotoxicity and exacerbates neurotoxicity (Barger et al., 2007), but also participate in its uptake by expressing different types of glutamate transporters (Kettenmann et al., 2011), as is the case of GLT-1 that is upregulated in activated microglia (Lopez-Redondo et al., 2000; Persson et al., 2005). Release of substantial levels of glutamate from microglia in response to bilirubin was previously demonstrated (Gordo et al., 2006; Silva et al., 2010). Here, GLT-1 increase only occurred in the young/immature Bf-treated microglia suggesting a protective intervention against the Bf stimulus on glutamate release (Fernandes et al., 2004; Gordo et al., 2006). Actually, UCB-induced release of glutamate is higher in young than in old nerve cells (Falcão et al., 2006) and may relate with the increased levels of HMGB1, which are known to stimulate the glutamate release in mice synaptosomes and astrocytic gliosomes (Pedrazzi et al., 2006).

In earlier studies we have demonstrated that UCB induces nNOS expression and NO production in immature rat neurons cultured for 3DIV (Vaz et al., 2011b), while also causes NO production by activated microglia in organotypic hippocampal slices from 7- to 10-day-old Wistar rats (Silva et al., 2012).

In this study, we showed that Bf at 100 nM upregulates iNOS gene expression in both young and aged cultured cortical mouse microglia. Overexpression of iNOS was found in amoeboid microglia in developing retina, while downregulated levels were observed in ramified microglia (Sierra et al., 2014). Several signalling cascades determine the expression of iNOS in glial cells, whose elevated levels are observed during neuroinflammation in neurodegenerative disorders (Saha and Pahan, 2006). Our data show that Bf produces a wide variety of proinflammatory and degenerative stimuli mainly in the young microglia and, therefore, iNOS upregulation may result from Bf direct immunostimulation. On the other hand, iNOS increase in 16DIV cells can be related with an enhanced IL-1β-induced iNOS due to microglia alterations by aging (Norden and Godbout, 2013), as the enhanced late apoptosis/necrosis suggest in these cells. However, just the 2DIV Bf-treated cells led to NO generation by either one of the tested concentrations, since the 16DIV microglia contrasted by the reduced production of NO. Thus, mitochondria nitrosative-induced stress was just observed in young microglia and by the Bf highest level. Actually mitochondria has been pointed as a target of BIND (Brites, 2012) and mitochondrial depolarization and impairment of the respiratory chain previously reported in UCB-treated immature neurons (Vaz et al., 2010).

Originally, it was accepted that microglia activation resulted in two main phenotypes, a proinflammatory M1 subtype and an anti-inflammatory and reparative subtype M2, though more recent studies showed that M1 and M2 are not isolated phenomena (Ransohoff, 2016) and that multiple microglia phenotypic markers may co-exist (Torres-Platas et al., 2014). Evidences show that microglia can switch phenotypes and differentially express gene signatures in response to a variety of insults, either using microglial cell lines or primary cultures from mice modeling amyotrophic lateral sclerosis (ALS) and Alzheimer’s disease (AD) (Caldeira et al., 2017; Pinto et al., 2017; Vaz et al., 2019). Here, we demonstrate that Bf increases the expression of mRNA levels of MHCII, TNF-α, IL-1β, and IL-6 inflammatory mediators in 2DIV microglia, from which only IL-1β was found elevated in 16DIV cells. These findings point to a predominant switch of young microglia to a pro-inflammatory phenotype by Bf, while it only scarcely activated the aged cells. Reinforcing the pro-inflammatory stimulus of Bf, downregulation of IL-10, an anti-inflammatory cytokine playing a critical role in immunoregulation (Saxena et al., 2015; Ip et al., 2017), was present in both cell aged types. In short, microglia may be primed by Bf during neonatal hyperbilirubinemia leading to neurodevelopmental critical outcomes and increased susceptibilities to cognitive deficits by a secondary inflammatory insult in later life (Hoeijmakers et al., 2016; Paolicelli and Ferretti, 2017). To the best of our knowledge, this the first study showing that immature/neonatal microglia have an increased vulnerability to the pro-inflammatory effects of elevated concentrations of Bf. The slow response of the 16DIV maturated microglia to Bf immunostimulation may be associated to an acquired senescent-like phenotype (Caldeira et al., 2014) that was later demonstrated to be irresponsive to Aβ insult (Caldeira et al., 2017). Interestingly, as here reported, elevation of IL-1β was produced in 2DIV and 16DIV microglia upon Aβ incubation. Besides its role as a proinflammatory cytokine, IL-1β also belongs to the senescent-associated secretory phenotype (SASP) proteins (Ozcan et al., 2016). Thus, we hypothesize that the increased IL-1β mRNA levels in long maturated cells exposed to Bf may derive from the cell ageing, instead of being exclusively linked to a pro-inflammatory state. Up-regulation of arginase 1, mainly associated with the resolution of damage and associated with a neuroprotective profile (Colton, 2009) was similarly observed in cells cultured for short or long time after Bf treatment. Indeed, compensatory immune-inflammatory responses to UCB have been proposed in patients with bilirubin encephalopathy, where certain bioactive proteins, including defensins (e.g. α- defensin 1, DEFA1) and/or alarmins (e.g. C−reactive protein, S100A7 or S100A9) were differentially up/-down-regulated in extracellular vesicles isolated from the cerebrospinal fluid of those patients (Tan et al., 2020). Nevertheless, arginase 1 was also shown to support the inflammatory state in some studies (Abad Dar and Holscher, 2018) and to be upregulated by LPS in others (Zhang et al., 2009). To additionally refer that upregulation of arginase 1 in both 2DIV and 16DIV microglia was observed upon Aβ incubation, as well (Caldeira et al., 2017).

Considering the key roles of microglia in neurogenesis and developmental synaptic pruning, as well as their trophic support (Cowan and Petri, 2018), the early-life induced inflammation by Bf may have immediate and lasting consequences. Despite knowledge about miRNAs in neuroinflammation (Karthikeyan et al., 2016), little is known on their role in microglia inflammatory responses and mediated pathologies, though their dysregulation has been associated to perinatal brain injury (Cho et al., 2019). To date, no study has explored if and how inflamma-miRs are dysregulated in microglia activated by bilirubin, and whether the signature differs according to cell maturation. We assessed the expression of a set of inflamma-miRs recognized as mediators of the immunoregulatory microglial functions (Fernandes et al., 2018; Vaz et al., 2019), namely miR-155, miR-146a, miR-125b, miR-124, and miR-21. Validating the Bf-immunostimulant effect on young microglia pro-inflammatory state, all miRNAs were found upregulated except miR-124 that mitigates microglia-induced neuroinflammation (Yu et al., 2017). From these miR-155 was shown to induce a pro-inflammatory response and to be early upregulated in the triple transgenic AD mouse model, while miR-124 acts as a negative regulator (Ponomarev et al., 2011; Guedes et al., 2014; He et al., 2014). Attesting the heterogeneity of activated microglia phenotypes by Bf in 2DIV cells, the overexpression of miR-21 and miR-146a shows that microglia may also induce the production of these inflammatory regulators (Wang et al., 2018) subsequently to Bf-induced microglial immunostimulation and upregulation of miR-155. Another important fine tuner of inflammation is miR-125b related to microglia activation in ALS (Parisi et al., 2016). As a curiosity, in obstructive jaundice, miR-21 and miR-125b, among others, were found upregulated in the mouse liver (Kanda et al., 2010) and the later associated with increased tau hyperphosphorylation (Banzhaf-Strathmann et al., 2014; Zhang et al., 2019). Again, exposure to bilirubin early in life in newborn rats was suggested to promote AD-like pathological changes later in life, including tau protein hyperphosphorylation, Aβ production and spatial learning and memory injuries (Chen et al., 2019), pointing to the significance of microglial priming in jaundiced infants during the neonatal period.

In contrast with the early activation of cortical microglia by Bf concentrations of 50 nM and 100 nM, decrease of miR-155, miR-146a, and miR-124, the three miRNAs most recognized as being involved in microglia inflammatory responses (Su et al., 2016), in the aged cells highlight either a senescence-like cell behaviour and irresponsiveness to Bf stimulus, or alternatively an adaptive response to counteract the inflammatory stimulus (Tili et al., 2007; Caldeira et al., 2014; Parisi et al., 2016; Caldeira et al., 2017).

**Figure 8:**
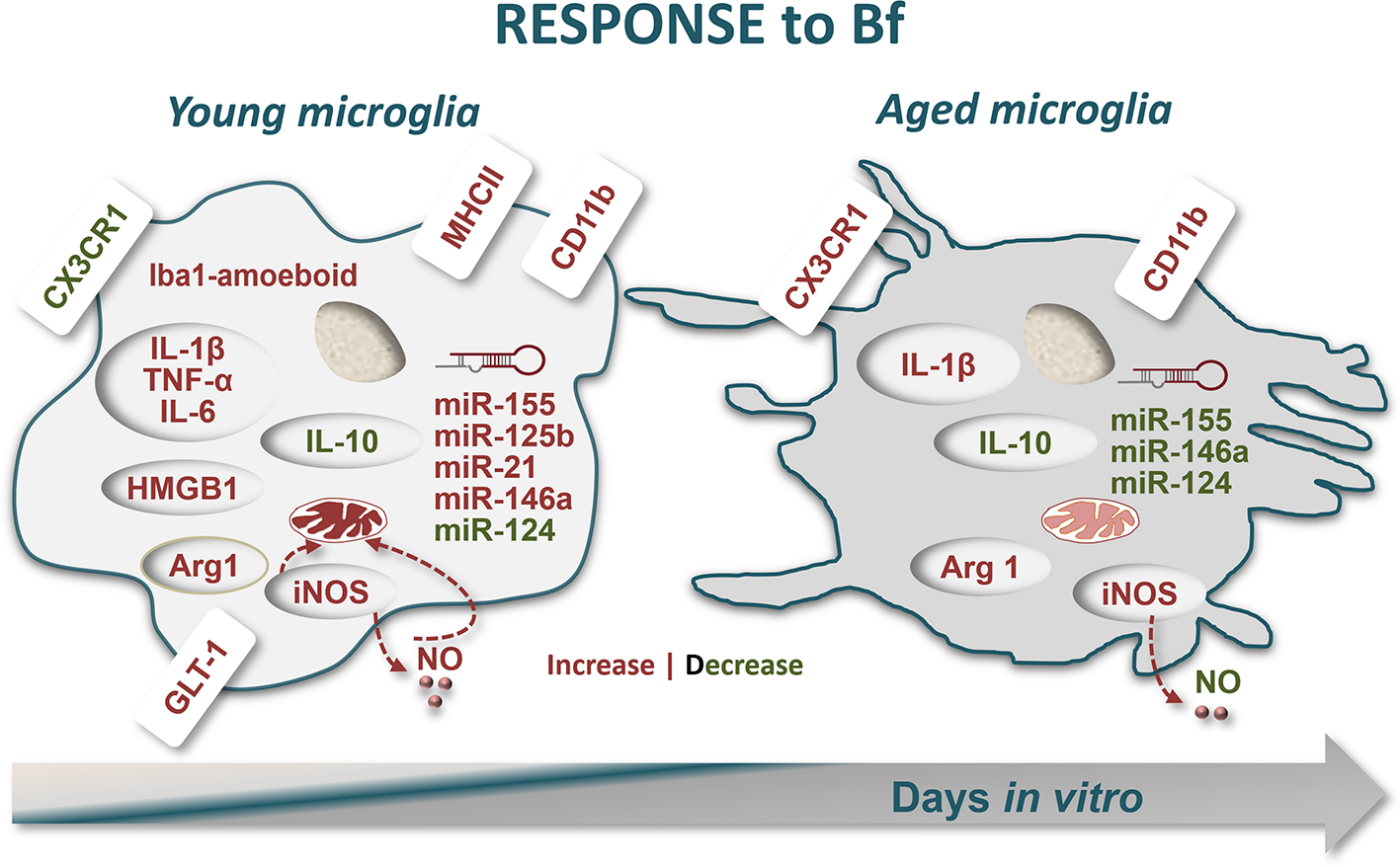
Schematic representation of microglia response to free bilirubin (Bf), considering young and aged cultured cells. Microglial cells were treated at 2 (young) and 16 days-in-vitro (DIV) (aged) with 50 nM and 100 nM Bf for 24 h. Results show that short-cultured 2DIV microglia are more reactive to Bf than the long-cultured 16DIV cells. In young cells, both Bf concentrations favour Iba-1 amoeboid microglia and cause ~1.5-fold increase in CD11b-positive cells over aged microglia. Low levels of CX3CR1 and increased values of the alarmin HMGB1 in Bf-treated young cells, not exhibited by the aged cells, reinforce their proneness to Bf stimulation, a finding also reproduced by GLT-1 upregulation. Bf-induced mitochondrial-nitrosative stress (increased levels of either iNOS and NO) is again more notorious in 2DIV cells. These young microglia reveal to be unique in overexpressing inflammatory-associated markers upon Bf interaction (MHCII/TNF-α/IL1β/IL-6), apart arginase 1 (Arg1) and IL-1β that also increase in aged cells. The immunostimulant effect of Bf in the neonatal-like microglia and their increased reactivity to the injury is translated in the elevation of most of the inflammatory-associated miRNAs in 2DIV cells (miR-155/-125b/-21/-146a), while a depressed signature is found in the aged microglia. However, a common finding in the differently aged microglia by Bf interaction was the reduction of the “calming” IL-10 and miR-124 mediators in both, thus indicating the presence of shared immune responses. In sum, immune activation by Bf is more robust in early-life microglia and may increasingly impact on neonatal illness by hyperbilirubinemia, thus requiring immunoregulatory approaches to prevent clinical deterioration and related neurodevelopmental disabilities.

In conclusion, this study adds to our understanding on how Bf can affect microglia dynamic properties, and support the vulnerability of the young cells, which turn overactivated even at moderated levels of Bf, as schematically represented in **Figure 8**. The susceptibility of newborns and their immature nerve cells to BIND, together with the deregulation of microglial immune response by Bf with early and ensuing impacts in degenerative processes, suggest immunomodulation as a promising therapeutic strategy in neonates in risk for bilirubin encephalopathy and critical outcomes.

## Data Availability Statement

All data generated or analysed during this study are included in this published article (and its **Supplementary Information Files**).

## Ethics Statement

The animal study was reviewed and approved by Portuguese National Authority (General Direction of Veterinary).

## Author Contributions

DB conceived and directed the project. AV, AF and DB planned and designed the experiments. AV and AF performed most of the experimental work, analysed data and contributed to the writing of the manuscript. CS participated in the immunocytochemistry studies and ES contributed for the assays regarding mitochondrial viability. DB critically reviewed and wrote the final version of the manuscript in consultation with AV. All authors approved the submitted version.

## Conflict of Interest

The authors declare that the research was conducted in the absence of any commercial or financial relationships that could be construed as a potential conflict of interest.
