# Supplementary material for: Microglia Susceptibility to Free Bilirubin Is Age-Dependent
Source: Front Pharmacol. 2020 Jul 14;11:1012. doi: 10.3389/fphar.2020.01012 (PMC7381152; doi:10.3389/fphar.2020.01012)
Supplement: Supplementary file 1 [file DataSheet_1.docx]

*Supplementary material*

Frontiers in Pharmacology

**Microglia Susceptibility to Free Bilirubin is Age-Dependent**

Vaz AR^1,2,&^, Falcão AS^1,2,3,&^, Scarpa E^1^, Semproni C^1^, Brites D^1,2^*

^1^Research Institute for Medicines (iMed.ULisboa), Faculty of Pharmacy, Universidade de Lisboa, Av. Professor Gama Pinto, 1649-003 Lisbon, Portugal

^2^Department of Biochemistry and Human Biology, Faculty of Pharmacy, Universidade de Lisboa, Lisbon, Portugal

^3^Chronic Diseases Research Center (CEDOC), Nova Medical School, Universidade Nova de Lisboa, Campo dos Mártires da Pátria 130, 1169‐056 Lisbon, Portugal

**^&^equal contribution; *Correspondence:** [dbrites@ff.ulisboa.pt](mailto:dbrites@ff.ulisboa.pt)

| **Gene** | **Forward primer sequence** | | | | **Reverse primer sequence** | | |
| --- | --- | --- | --- | --- | --- | --- | --- |
| ***CX3CR1*** | 5’-TCGTCTTCACGTTCGGTCTG-3’ | | | | 5’-CTCAAGGCCAGGTTCAGGAG-3’ | | |
| ***iNOS*** | 5’-ACCCACATCTGGCAGAATGAG-3’ | | | | 5’-AGCCATGACCTTTCGCATTAG-3’ | | |
| ***Arginase 1*** | 5’-CTTGGCTTGCTTCGGAACTC-3’ | | | | 5’-GGAGAAGGCGTTTGCTTAGTTC-3’ | | |
| ***HMGB1*** | 5’-CTCAGAGAGGTGGAAGACCATGT-3’ | | | | 5’-GGGATGTAGGTTTTCATTTCTCTTTC-3’ | | |
| ***MHCII*** | 5’-TGGGCACCATCTTCATCATTC-3’ | | | | 5’- GGTCACCCAGCACACCACTT-3’ | | |
| ***IL-1β*** | 5'- CAGGCTCCGAGATGAACAAC-3' | | | | 5'- GGTGGAGAGCTTTCAGCTCATA-3' | | |
| ***IL-6*** | 5’-CCGGAGAGAGGAGACTTCACAG-3’ | | | | 5’-GGAAATTGGGGTAGGAAGGA-3’ | | |
| ***IL-10*** | 5’-CCAGTTTTACCTGGTAGAAGTGAG-3’ | | | | 5’-TGTCTAGGTCCTGGAGTCCAGCAGACTC-3’ | | |
| ***TNF-α*** | 5’-TACTGAACTTCGGGGTGATTGGTCC-3’ | | | | 5’-CAGCCTTGTCCCTTGAAGAGAACC-3’ | | |
| ***β-actin*** | 5’-GCTCCGGCATGTGCAA-3’ | | | | 5’-AGGATCTTCATGAGGTAGT-3’ | | |
| **miRNA** | | | **Target sequence** |  | | |  |
| **hsa-miR-146a-5p** | | 5’-UGAGAACUGAAUUCCAUGGGUU-3’ | | | |  | |
| **hsa-miR-21-5p** | | 5’-UAGCUUAUCAGACUGAUGUUGA-3’ | | | |  | |
| **mmu-miR-155-5p** | | 5’-UUAAUGCUAAUUGUGAUAGGGGU-3’ | | | |  | |
| **hsa-miR-125b-5p** | | 5’-UCCCUGAGACCCUAACUUGUGA-3’ | | | |  | |
| **hsa-miR-124-3p** | | 5’-UAAGGCACGCGGUGAAUGCC-3’ | | | |  | |
| **SNORD110** | | Reference gene | | | |  | |

**Supplementary Table 1: List of primer sequences used in qRT-PCR**
